# Detection of human bocavirus in Saudi healthy blood donors

**DOI:** 10.1371/journal.pone.0193594

**Published:** 2018-02-28

**Authors:** Ahmed S. Abdel-Moneim, Mohammad E. Mahfouz, Dalia M. Zytouni

**Affiliations:** 1 Department of Microbiology, College of Medicine, Taif University, Al-Taif, Saudi Arabia; 2 Virology Department, Faculty of Veterinary Medicine, Beni-Suef University, Beni-Suef, Egypt; 3 Department of Surgery, College of Medicine, Taif University, Al-Taif, Saudi Arabia; 4 King Faisal Hospital, Al-Taif, Saudi Arabia; Kliniken der Stadt Köln gGmbH, GERMANY

## Abstract

Human bocavirus is associated with respiratory disease worldwide, mainly in children. There are conflicting results, however, regarding the existence of the HBoV in blood donors. Three hundred whole blood samples from non-immunodeficient healthy blood donors were screened for the presence of HBoV by polymerase chain reaction. The HBoV genotype of positive samples was determined using direct gene sequencing. Twenty-one out of the three hundred blood samples were found to be positive for HBoV. Sequence analysis of the positive samples revealed that all the strains were related to the HBoV-1 type with a low rate of variation among the detected sequences. It was concluded that there is a considerable risk of contracting HBoV from a blood transfusion from normal healthy individuals.

## Introduction

Human bocavirus (HBoV) is a parvovirus, discovered in 2005, that is frequently associated with respiratory tract infection in young children [1]. There are four different types of HBoV (1–4), HBoV-1 and HBoV-3, which belong to *Primate bocaparvovirus* 1, and HBoV-2a-c and HBoV-4, which are members of the *Primate bocaparvovirus* 2 species [2]. Based on the full genome, HBoV-1 viruses are clustered into three main lineages: A to C, with lineage A further subdivided into three sub-lineages, A1–A3 based on the full length VP1/2 gene [3].

Polymerase chain reaction (PCR) is the most common method used for the detection of bocavirus. HBoV-1 is spread globally and is present in between 3 and 22% of examined nasopharyngeal samples. Infection is mainly detected in children under five years with respiratory tract illness [4–6]. HBoV DNA has also been detected in acute- and convalescent-phase sera from children with acute respiratory tract infections [5]. It is infrequently detected in adults, however [7, 8]. There are conflicting results regarding the existence of the HBoV in blood. Three studies did not detect HBoV-1 DNA in blood donors or plasma-derived medicinal products [9–11], but some other studies have detected HBoV DNA in healthy blood donors and in blood products [12, 13].

Many countries including the USA, Canada, and Japan as well as many European countries, have currently, strengthened their hemovigilance systems to identify new pathogens that constitute potential transfusion risks. Currently, however, the epidemiological data about the existence of HBoV in blood is scarce worldwide. Accordingly, the current study was intended to screen for the existence of HBoV DNA in healthy Saudi blood donors.

## Materials and methods

### Ethical approval

Both King Faisal Hospital and Taif University ethical committees approved the study. Informed written consent was obtained from the voluntary blood donors.

### Samples

Three hundred whole blood samples from Saudi nationals (80 female and 220 male) with an age range of 20–48 years old, were screened for the presence of HBoV DNA. The blood samples were collected from voluntary donors in Taif, Saudi Arabia, during January 2016 and December 2016, King Faisal Hospital, Al-Taif, Saudi Arabia. The inclusion criteria included healthy blood donors who showed negative results for the common bloodborne pathogens including HBV, HCV, HIV and HTLV using Bio-Rad ELISA kits as routinely conducted in the blood bank, King Faisal Hospital, Al-Taif Saudi Arabia. Individuals who showed positive results to any of the previously mentioned viruses, or immunocompromised patients, have been excluded from the study.

### Polymerase chain reaction

Genomic DNA was extracted from 200 μL of whole blood using a DNA extraction kit (Thermo Scientific, Wilmington, DE) according to the manufacturer’s instructions. Samples were screened for the presence of the HBoV using conventional polymerase chain reaction (PCR) assay using a modified protocol of the previously described in [14, 15]. The following primer set that flanks a 378-bp a highly conserved sequence in the terminal 1/10 of the VP1 gene 3’ and the last 1/4 of the 3’of the non-coding region were used:VP/NC For 5’-AGCTGTGAGATTGTATGGGAAG-3’ and PanBoca Rev: 5’-AAAACAGCTCCCCCCACAAT-3’. The amplification was conducted using PCR mastermix (Solis BioDyne, Inc., Estonia). The thermal cycler conditions were 95°C for 5 min and 35 cycles at 94°C for 30 s, 50°C for 40 s and 72°C for 45 sec. This was followed by a final elongation step at 72 °C for 10 min. DNA extracted from Eg/BSU-1, a previously characterized strain from Egyptian children [15], was used as the positive control in all reactions. The PCR amplicons were analysed by electrophoresis in 2% agarose gels.

### HBoV real-time PCR

Samples that were positive according to PCR were screened quantitatively using an HBoV real-time kit (Life River, Shanghai, China) according to the manufacturer’s instructions in Eppendorf Mastercycler^®^ ep realplex2.

### Sequencing and sequence analysis

DNA was extracted from the gel bands of the PCR amplicons using Wizard SV Gel extraction kit (Promega, UK). Direct gene sequencing was conducted commercially in both forward and reverse directions using the same primer set as was used in the PCR amplification. MEGA 5.2 software was used to process the raw sequence. Human HBoV sequences from different types were used in the Multisequence alignment was performed using the processed sequences (MF977917-MF977937) as well as representative HBoV strains from different HBoV types available in the GenBank database. Phylogenetic analysis was conducted using MEGA, version 5.2. The phylogeny was performed using the maximum likelihood protocol with 1000 bootstrap replications, Tamura-Nei model with gap deletions and a uniform rate among sites. The tree interface option included ML heuristic method and the nearest neighbor-interchange (NNI).

## Results and discussion

Twenty-one out of 300 samples (7%) showed a positive reaction HBoV. All positive samples were confirmed by direct gene sequence of the amplified gene product. The positive samples possessed low virus concentrations (≥-10^3^ copies/ml) as evidenced by real-time PCR of the positive. All subjects were without any symptoms or clinical signs. In comparison, parvovirus, B19V, is allowed in blood pools used for blood products, as long as its concentration is below 10^4^ IU/ml, as reviewed in [4]. This is on the basis that the neutralizing antibodies in the donor will also be protective for the recipient provided that the viral load is low and in the absence of underlying diseases in the recipient [16]. To the best of our knowledge no current regulation restrict the HBoV positive blood or blood products.

Our findings support those other studies, in Italy and China, that have detected HBoV DNA in healthy blood donors and in blood [12, 13]. HBoV was reported in 5.5% of Italian healthy blood donors [12] and 9.1% of the healthy Chinese plasma donors [13]. The asymptomatic presence of the virus in a proportion of the population may be because HBoV is among the viruses that possibly induce a persistent infection in infected cells, mainly in the lymphocytic lineage [17], and because both persistence and reactivation are possible during HBoV infection [17–19]. This may lead to long-term virus shedding in the healthy population follow an asymptomatic infection during adulthood. Even assuming that HBoV induces a persistent infection and can be transmitted in the blood, however, this does not necessarily translate to a long-lasting detectable level of viral DNA, since when some of the Chinese blood donors with the highest plasma viral load were subsequently tracked, it was revealed that the HBoV cleared from their system [13]. Meanwhile, it cannot be excluded that the blood donors had a mild HBoV infection before their blood donations or may have been in the convalescence phase in which a viremia is still detectable.

In contrast, along with the fact that other studies of individual donors and medicinal products failed to detect HBoV DNA [9–11] a study of 167 plasma pools also showed an absence of the virus [20]. This was attributed to the fact that low individual donor levels of HBoV DNA would be so diluted as to become undetectable in pools containing several thousand donations [20–22]. The sensitivity of nucleic acid screening of pooled samples depends on the concentration of virus in the samples, in addition to the sensitivity of the test method used and inter assay variability. All of which may result in false negative results [13, 22–26]. Mismatching of the oligonucleotide set during the amplification may also be responsible for decreasing the limit of virus detection [27].

**Fig 1:**
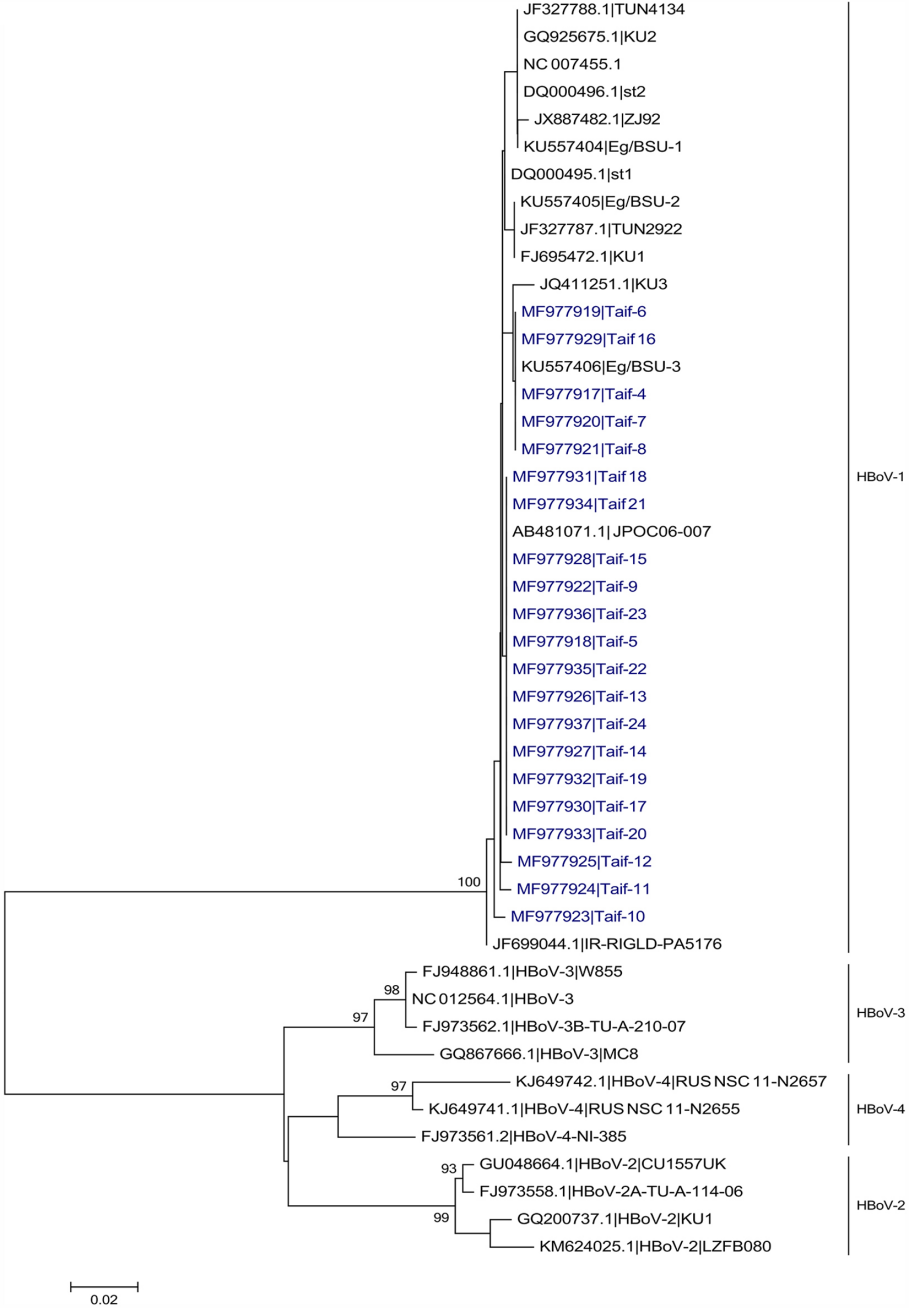
Phylogenetic tree of HBoV positive samples from healthy blood donors from Saudi Arabia. Maximum likelihood (ML) phylogenetic tree of HBoV strains detected in the current study (shown in blue) in comparison to different published HBoV strains.

The twenty-one HBoV-1 sequences in the current study were aligned with 25 reference sequences including representative strains from the four different HBoV viruses. The four different viruses of HBoV were clustered into four main branches, with bootstrap values of more than 90%. All the samples in the current study were clustered within the HBoV type 1 branch (Fig 1) with a low rate of nucleotide heterogeneity among different strains in the current study (S1 Fig). This result agrees with another study in China that also found also HBoV-1 to be the prevalent type in healthy plasma donors [13].

## Conclusion

In conclusion, the current study is the first to demonstrate and identify HBoV DNA in blood in Saudi blood donors. The possibility that HBoV could threaten blood safety requires further investigation.

## Supporting information

S1 Fig Deduced nucleotide sequence of the VP1/NC of HBoV-1 from Saudi blood donors in comparison to different HBoV published strains. Strains related to the HBoV-1 genotype are boxed. Representative strains from HBoV-2, HBoV-3 and HBoV-4 are included.
(PDF)
