# Correction: Detection of human bocavirus in Saudi healthy blood donors

**DOI:** 10.1371/journal.pone.0196884

**Published:** 2018-05-02

**Authors:** Ahmed S. Abdel-Moneim, Mohammad E. Mahfouz, Dalia M. Zytouni

There is an error in the citation. Author Mohammad E. Mahfouz appears incorrectly. The correct citation is: Abdel-Moneim AS, Mahfouz ME, Zytouni DM (2018) Detection of human bocavirus in Saudi healthy blood donors. PLoS ONE 13(2): e0193594. https://doi.org/10.1371/journal.pone.0193594
