# Supplementary material for: Detection of human bocavirus in Saudi healthy blood donors
Source: PLoS One. 2018 Feb 28;13(2):e0193594. doi: 10.1371/journal.pone.0193594 (PMC5831472; doi:10.1371/journal.pone.0193594)
Supplement: S1 Fig — Strains related to the HBoV-1 genotype are boxed. Representative strains from HBoV-2, HBoV-3 and HBoV-4 are included. (PDF) [file pone.0193594.s001.pdf]

Supplement 1: Deduced nucleotide sequence of the VP1/NC of HBoV-1 from Saudi blood donors in comparison to different HBoV published strains.

DQ000495.1|st1	AGCTGTGAAA	TTGTATGGGA	AGTAGAAAGA	TACGCAACAA	AGAACTGGCG	TCCAGAAAGA	AGACATACTG	CACTCGGGAT
FJ695472.1|KU1	....	....	....	....	....	....	....	....
DQ000496.1|st2	....	....	G.........	....	....	....	....	....
NC_007455.1	....	....	G.........	....	....	....	....	....
GQ925675.1|KU2	....	....	G.........	....	....	....	....	....
JX887482.1|ZJ92	....	....	G.........	....	....	....	....	....
JF327788.1|TUN4134	....	....	G.........	....	....	....	....	....
KU557404|Eg/BSU-1	....	....	G.........	....	....	....	....	....
JQ411251.1|KU3	........G.	....	....A.....	....	....	....	....	....
JF327787.1|TUN2922	....	....	....	....	....	....	....	....
KU557405|Eg/BSU-2	....	....	....	....	....	....	....	....
KU557406|Eg/BSU-3	........G.	....	....	....	....	....	....	....
AB481071.1|_JPOC06-007	........G.	....	....	....	....	....	....	....
JF699044.1|IR-RIGLD-PA5176	........G.	....	....	....	....	....	....	....T.....
MF977917|Taif-4	........G.	....	....	....	....	....	....	....
MF977918|Taif-5	........G.	....	....	....	....	....	....	....
MF977919|Taif-6	........G.	....	....	....	....	....	....	....
MF977920|Taif-7	........G.	....	....	....	....	....	....	....
MF977921|Taif-8	........G.	....	....	....	....	....	....	....
MF977922|Taif-9	........G.	....	....	....	....	....	....	....
MF977923|Taif-10	........G.	....	....	....	....	....	....	....
MF977924|Taif-11	........G.	....	....	....	....	....	....	....
MF977925|Taif-12	........G.	....	....	....	....	....	....	....
MF977926|Taif-13	........G.	....	....	....	....	....	....	....
MF977927|Taif-14	........G.	....	....	....	....	....	....	....
MF977928|Taif-15	........G.	....	....	....	....	....	....	....
MF977929|Taif_16	........G.	....	....	....	....	....	....	....
MF977930|Taif_17	........G.	....	....	....	....	....	....	....
MF977931|Taif_18	........G.	....	....	....	....	....	....	....
MF977932|Taif_19	........G.	....	....	....	....	....	....	....
MF977933|Taif-20	........G.	....	....	....	....	....	....	....
MF977934|Taif_21	........G.	....	....	....	....	....	....	....
MF977935|Taif_22	........G.	....	....	....	....	....	....	....
MF977936|Taif-23	........G.	....	....	....	....	....	....	....
MF977937|Taif-24	........G.	....	....	....	....	....	....	....
GU048664.1|HBoV-2|CU1557UK	.....C....	....C.....	...T......	....	.........A	A....G....	.....C..C.	....T..TC.
GQ200737.1|HBoV-2|KU1	.....C....	....C.....	...T......	....	.........A	A.........	.....C..C.	....T..TC.
KM624025.1|HBoV-2|LZFB080	.....C....	....C.....	...T......	....	.........A	A....G....	.....C..C.	.G..T..TC.
FJ973558.1|HBoV2A-TU-A-114-06	.....C....	....C.....	...T......	....	.........A	A....G....	.....C..C.	....T..TC.
GQ867666.1|HBoV-3|MC8	.....C....	....C.....	...T......	..T.......	.........A	A.........	.....C..A.	.......CC.
gFJ948861.1|HBoV-3|W855	.....C....	....C.....	...C......	..T.......	.........A	A.........	.....C..A.	.......CC.
FJ973562.1|HBoV3B-TU-A-210-07	.....C....	....C.....	...C......	..T.......	.........A	A.........	.....C..A.	.......CC.
NC_012564.1|HBoV-3	.....C....	....C.....	...C......	..T.......	.........A	A.........	.....C..A.	.......CC.
KJ649742.1|HBoV-4|RUS_NSC_11-N2657	.....C....	....C.....	...T......	..T.......	.........A	A.........	.....C..AA	..T.T..TC.
FJ973561.2|HBoV-4-NI-385	....	....C.....	...T......	..T.......	.........A	A.........	.....C..AA	..T.T..TC.
KJ649741.1|HBoV-4|RUS_NSC_11-N2655	.....C....	....C.....	...T......	..T.......	.........A	A.........	.....C..AA	..T.T..TC.

Supplement 1 continue

```
DQ000495.1|st1                  GTCACTGGGA GGAGAGAGCA ACTACACGCC TACATACCAC GTGGATCCAA CAGGAGCATA CATCCAGCCC ACGTCATATG
FJ695472.1|KU1                  .......... .......... .......... .......... .......... .......... .......... ..........
DQ000496.1|st2                  .......... .......... .......... .......... .......... .......... .......... ..........
NC_007455.1                     .......... .......... .......... .......... .......... .......... .......... ..........
GQ925675.1|KU2                  .......... .......... .......... .......... .......... .......... .......... ..........
JX887482.1|ZJ92                 .......... .......... .......... .......... .......... .......... .......... ..........
JF327788.1|TUN4134              .......... .......... .......... .......... .......... .......... .......... ..........
KU557404|Eg/BSU-1               .......... .......... .......... .......... .......... .......... .......... ..........
JQ411251.1|KU3                  .......... .....A.... .......... .......... .......... .......... .......... ..........
JF327787.1|TUN2922              .......... .......... .......... .......... .......... .......... .......... ..........
KU557405|Eg/BSU-2               .......... .......... .......... .......... .......... .......... .......... ..........
KU557406|Eg/BSU-3               .......... .......... .......... .......... .......... .......... .......... ..........
AB481071.1|_JPOC06-007          .......... .......... .......... .......... .......... .......... .......... ..........
JF699044.1|IR-RIGLD-PA5176      .......... .......... .......... .......... .......... .......... .......... ..........
MF977917|Taif-4                 .......... .......... .......... .......... .......... .......... .......... ..........
MF977918|Taif-5                 .......... .......... .......... .......... .......... .......... .......... ..........
MF977919|Taif-6                 .......... .......... .......... .......... .......... .......... .......... ..........
MF977920|Taif-7                 .......... .......... .......... .......... .......... .......... .......... ..........
MF977921|Taif-8                 .......... .......... .......... .......... .......... .......... .......... ..........
MF977922|Taif-9                 .......... .......... .......... .......... .......... .......... .......... ..........
MF977923|Taif-10                .......... .......... .......T.. .......... .......... .......... .......... ..........
MF977924|Taif-11                .......... .......... .......... .......... .......... .......... .......... ..........
MF977925|Taif-12                .......... .......... .......... .......... .......... .......... .......... ..........
MF977926|Taif-13                .......... .......... .......... .......... .......... .......... .......... ..........
MF977927|Taif-14                .......... .......... .......... .......... .......... .......... .......... ..........
MF977928|Taif-15                .......... .......... .......... .......... .......... .......... .......... ..........
MF977929|Taif_16                .......... .......... .......... .......... .......... .......... .......... ..........
MF977930|Taif-17                .......... .......... .......... .......... .......... .......... .......... ..........
MF977931|Taif_18                .......... .......... .......... .......... .......... .......... .......... ..........
MF977932|Taif-19                .......... .......... .......... .......... .......... .......... .......... ..........
MF977933|Taif-20                .......... .......... .......... .......... .......... .......... .......... ..........
MF977934|Taif_21                .......... .......... .......... .......... .......... .......... .......... ..........
MF977935|Taif-22                .......... .......... .......... .......... .......... .......... .......... ..........
MF977936|Taif-23                .......... .......... .......... .......... .......... .......... .......... ..........
MF977937|Taif-24                .......... .......... .......... .......... .......... .......... .......... ..........
GU048664.1|HBoV-2|CU1557UK      TGG.A.C... .....AGAA. ..ATA.AT.. A..T.....T ..A..CAA.. AT...AA... ...T.....A ..AA...GG.
GQ200737.1|HBoV-2|KU1           TGG.A.T... .....AGAA. ..GTA.AT.. A..T..T..T ..A..CAA.. AT...AA... ...T..A..A ..AA.T.GG.
KM624025.1|HBoV-2|LZFB080       TGG.A.T... .....AGAA. ..ATA.AT.. A..C..T..T ..A..CAA.. AT...AA... ...T..A..A ..AA.T.GG.
FJ973558.1|HBoV2A-TU-A-114-06   TGG.A.C... .....AGAA. ..ATA.AT.. A..T.....T ..A..CAA.. AT...AA... ...T.....A ..AA...GG.
GQ867666.1|HBoV-3|MC8           TGG.A.T... ....CAGATG .AATT.AC.. A......... ..T..CAA.. AT....TC.. ...T..A..T ..AA...GG.
gFJ948861.1|HBoV-3|W855         TGG.A.T... ..G.CAGATG .AAT..AC.. A........T ..T..CAA.. AC........ ...T..A..T ..AA...GG.
FJ973562.1|HBoV3B-TU-A-210-07   TGG.A.T... ..G.CAGATG .AAT..AC.. A........T ..T..CAA.. AC........ ...T..A..T ..AA...GG.
NC_012564.1|HBoV-3              TGG.A.T... ..G.CAGATG .AAT..AC.. A........T ..T..CAA.. AC........ ...T..A..T ..AA...GG.
KJ649742.1|HBoV-4|RUS_NSC_11-N2657 TGG.A.T... ....CTGA.. ...TA.AT.. A..C.....T ..T..CAA.. AC....TT.. ...T..A..T ..AA...GG.
FJ973561.2|HBoV-4-NI-385        TGG.A.T... ....CTGA.. ...TA.AT.. A..C.....T ..T..CAA.. AC...A.T.. ...T..A..A ..AA...GG.
KJ649741.1|HBoV-4|RUS_NSC_11-N2655 TGG.A.T... ....CTGA.. ...TA.AT.. A..C.....T ..T..CAA.. AC....TT.. ...T..A..T ..AA...GG.
```

Supplement 1 continue

```
DQ000495.1|st1                       ATCAGTGTAT GCCAGTAAAA ACAAACATCA ATAAAGTGTT GTAATCTTAT AAGCCTCTTT TTTGCTTCTG CTTACAAGTT
FJ695472.1|KU1                       ....A..... .......... .......... .......... .......... .......... .......... ..........
DQ000496.1|st2                       .......... .......... .......... .......... .......... .......... .......... ..........
NC_007455.1                          .......... .......... .......... .......... .......... .......... .......... ..........
GQ925675.1|KU2                       .......... .......... .......... .......... .......... .......... .......... ..........
JX887482.1|ZJ92                      .......... .......G.. .......... .......... .......... .......... .......... ..........
JF327788.1|TUN4134                   .......... .......... .......... .......... .......... .......... .......... ..........
KU557404|Eg/BSU-1                    .......... .......... .......... .......... .......... .......... .......... ..........
JQ411251.1|KU3                       ....A..... .......... .......... .......... .......... .......... .......... ..........
JF327787.1|TUN2922                   ....A..... .......... .......... .......... .......... .......... .......... ..........
KU557405|Eg/BSU-2                    ....A..... .......... .......... .......... .......... .......... .......... ..........
KU557406|Eg/BSU-3                    ....A..... .......... .......... .......... .......... .......... .......... ..........
AB481071.1|_JPOC06-007               .......... .......... .......... .......... .......... .......... .......... ..........
JF699044.1|IR-RIGLD-PA5176           .......... .......... .......... .......... .......C.. .......... .......... ..........
MF977917|Taif-4                      ....A..... .......... .......... .......... .......... .......... .......... ..........
MF977918|Taif-5                      .......... .......... .......... .......... .......... .......... .......... ..........
MF977919|Taif-6                      ....A..... .......... .......... .......... .......... .......... .......... ..........
MF977920|Taif-7                      ....A..... .......... .......... .......... .......... .......... .......... ..........
MF977921|Taif-8                      ....A..... .......... .......... .......... .......... .......... .......... ..........
MF977922|Taif-9                      .......... .......... .......... .......... .......... .......... .......... ..........
MF977923|Taif-10                     .......... .......... .......... .......... .......... .......... .......... ..........
MF977924|Taif-11                     .......... .......... .......... .......... .......... .......... .......T.. ..........
MF977925|Taif-12                     .......... .......... .......... .......... .......... .......... .......... ..........
MF977926|Taif-13                     .......... .......... .......... .......... .......... .......... .......... ..........
MF977927|Taif-14                     .......... .......... .......... .......... .......... .......... .......... ..........
MF977928|Taif-15                     .......... .......... .......... .......... .......... .......... .......... ..........
MF977929|Taif_16                     ....A..... .......... .......... .......... .......... .......... .......... ..........
MF977930|Taif-17                     .......... .......... .......... .......... .......... .......... .......... ..........
MF977931|Taif_18                     .......... .......... .......... .......... .......... .......... .......... ..........
MF977932|Taif-19                     .......... .......... .......... .......... .......... .......... .......... ..........
MF977933|Taif-20                     .......... .......... .......... .......... .......... .......... .......... ..........
MF977934|Taif_21                     .......... .......... .......... .......... .......... .......... .......... ..........
MF977935|Taif-22                     .......... .......... .......... .......... .......... .......... .......... ..........
MF977936|Taif-23                     .......... .......... .......... .......... .......... .......... .......... ..........
MF977937|Taif-24                     .......... .......... .......... .......... .......... .......... .......... ..........
GU048664.1|HBoV-2|CU1557UK           .CAT...CTA T..TA.C... .......... .......... .....A.CT. ......G..C A......A.. ....T.....
GQ200737.1|HBoV-2|KU1                .CAT...CTA T..TA.C... .......... .......... .......CT. ......G..C A......A.. ....T.....
KM624025.1|HBoV-2|LZFB080            .CAT...CTA T..TA.C... .......... .......... .......CT. ......G..C A......A.. ....T.....
FJ973558.1|HBoV2A-TU-A-114-06        .CAT...CTA T..TA.C... .......... .......... .......CT. ......A..C A......A.. ....T.....
GQ867666.1|HBoV-3|MC8                .CAT...CT. T.....T... .......... .......... .......CT. .......... A......AC. ...GT.....
gFJ948861.1|HBoV-3|W855              .CAT...CT. T.....T... .......... .......... .......CT. .......... A......AC. ...GT.....
FJ973562.1|HBoV3B-TU-A-210-07        .CAT...CT. T.....T... ........T. .......... .......CT. .......... A......AC. ...GT.....
NC_012564.1|HBoV-3                   .CAT...CT. T.....T... .......... .......... .......CT. .......... A......AC. ...GT.....
KJ649742.1|HBoV-4|RUS_NSC_11-N2657   .CAT...CT. T.....T... .......... .......... .....T.CT. .......... A......AC. ....T.....
FJ973561.2|HBoV-4-NI-385             .CAT...CT. T.....T... .......... .......... ....C...C. .......... .......A.. ....T.....
KJ649741.1|HBoV-4|RUS_NSC_11-N2655   .CAT...CT. T.....T... .......... .......... .....T.CT. .......... A......AC. ....T.....
```

Supplement 1 continue

```
DQ000495.1|st1                        CCTCCTCAAT GGACAAGCGG AAAGTGAAGG GTGACTGTAG TCCTGAGCTC ATGGGTTCAA GACCACAGCC CGATGGTAGT
FJ695472.1|KU1                        .......... .......... .......... .......... .......... .......... .......... ..........
DQ000496.1|st2                        .......... .......... .......... .......... .......... .......... .......... ..........
NC_007455.1                           .......... .......... .......... .......... .......... .......... .......... ..........
GQ925675.1|KU2                        .......... .......... .......... .......... .......... .......... .......... ..........
JX887482.1|ZJ92                       .......... .......... .......... .......... .......... .......... .......... ..........
JF327788.1|TUN4134                    .......... .......... .......... .......... .......... .......... .......... ..........
KU557404|Eg/BSU-1                     .......... .......... .......... .......... .......... .......... .......... ..........
JQ411251.1|KU3                        .......... .......... .......... .......... .......... .......... .......... ..........
JF327787.1|TUN2922                    .......... .......... .......... .......... .......... .......... .......... ..........
KU557405|Eg/BSU-2                     .......... .......... .......... .......... .......... .......... .......... ..........
KU557406|Eg/BSU-3                     .......... .......... .......... .......... .......... .......... .......... ..........
AB481071.1|_JPOC06-007                .......... .......... .......... .......... .......... .......... .......... ..........
JF699044.1|IR-RIGLD-PA5176            .......... .......... .......... .......... .......... .......... .......... ..........
MF977917|Taif-4                       .......... .......... .......... .......... .......... .......... .......... ..........
MF977918|Taif-5                       .......... .......... .......... .......... .......... .......... .......... ..........
MF977919|Taif-6                       .......... .......... .......... .......... .......... .......... .......... ..........
MF977920|Taif-7                       .......... .......... .......... .......... .......... .......... .......... ..........
MF977921|Taif-8                       .......... .......... .......... .......... .......... .......... .......... ..........
MF977922|Taif-9                       .......... .......... .......... .......... .......... .......... .......... ..........
MF977923|Taif-10                      .......... .......... .......... .......... .......... .......... .......... ..........
MF977924|Taif-11                      .......... .......... .......... .......... .......... .......... .......... ..........
MF977925|Taif-12                      .......... .......... .......... .........C .......... .......... .......... ..........
MF977926|Taif-13                      .......... .......... .......... .......... .......... .......... .......... ..........
MF977927|Taif-14                      .......... .......... .......... .......... .......... .......... .......... ..........
MF977928|Taif-15                      .......... .......... .......... .......... .......... .......... .......... ..........
MF977929|Taif_16                      .......... .......... .......... .......... .......... .......... .......... ..........
MF977930|Taif-17                      .......... .......... .......... .......... .......... .......... .......... ..........
MF977931|Taif_18                      .......... .......... .......... .......... .......... .......... .......... ..........
MF977932|Taif-19                      .......... .......... .......... .......... .......... .......... .......... ..........
MF977933|Taif-20                      .......... .......... .......... .......... .......... .......... .......... ..........
MF977934|Taif_21                      .......... .......... .......... .......... .......... .......... .......... ..........
MF977935|Taif-22                      .......... .......... .......... .......... .......... .......... .......... ..........
MF977936|Taif-23                      .......... .......... .......... .......... .......... .......... .......... ..........
MF977937|Taif-24                      .......... .......... .......... .......... .......... .......... .......... ..........
GU048664.1|HBoV-2|CU1557UK            ....TC.... .......A.. ....AA.... .........A ...C...... ...A....G. .G.T....T. ......C...
GQ200737.1|HBoV-2|KU1                 ....TC.... .......A.. ....AA.... .........A ...C...... ...A....G. .G.T....T. ......C...
KM624025.1|HBoV-2|LZFB080             ....TC.... .......A.. ....AA.... .........A ...C...... ..TA....G. .G.T.T..T. ......C...
FJ973558.1|HBoV2A-TU-A-114-06         ....TC.... .......A.. ....AA.... .........A ...C...... ...A....G. .G.T....T. ......C...
GQ867666.1|HBoV-3|MC8                 ....TC.... .......T.. ....AA.... .........A ...C...... .C.A....G. .G.T....T. ......C...
gFJ948861.1|HBoV-3|W855               ....TC.... A......T.. ....AA.... .........A ...C...... ...A....G. .G.T....T. ......C...
FJ973562.1|HBoV3B-TU-A-210-07         ....TC.... .......T.. ....AA.... .........A ...C...... ...A....G. .G.T....T. ......C...
NC_012564.1|HBoV-3                    ....TC.... .......T.. ....AA.... .........A ...C...... ...A....G. .G.T....T. ......C...
KJ649742.1|HBoV-4|RUS_NSC_11-N2657    ..C..C.... .......T.. ...ACCC... .........A ...C...... ...A....G. .GAT.GG.G. ......C...
FJ973561.2|HBoV-4-NI-385              ....TC.... .......T.. ....AA.... .........A ...C...... ...A....G. .G.T....T. ......C...
KJ649741.1|HBoV-4|RUS_NSC_11-N2655    ....TC.... .......T.. ...AAA.... .........A ...C...... ...A...... .G.T....T. ......C...
```

Supplement 1 continue

```
DQ000495.1|st1                    GGTGTTACCG TCTCGAACCT AGCCGACA-G CCCTTGTACA TTGTGGGGGG AGCTGTTTT
FJ695472.1|KU1                    .......... .......... ........-. .......... .......... .........
DQ000496.1|st2                    .......... .......... ........-. .......... .......... .........
NC_007455.1                       .......... .......... ........-. .......... .......... .........
GQ925675.1|KU2                    .......... .......... ........-. .......... .......... .........
JX887482.1|ZJ92                   .......... .......... ........-. .......... .......... .........
JF327788.1|TUN4134                .......... .......... ........-. .......... .......... .........
KU557404|Eg/BSU-1                 .......... .......... ........-. .......... .......... .........
JQ411251.1|KU3                    .......... .......... ........-. .......... .......... .........
JF327787.1|TUN2922                .......... .......... ........-. .......... .......... .........
KU557405|Eg/BSU-2                 .......... .......... ........-. .......... .......... .........
KU557406|Eg/BSU-3                 .......... .......... ........-. .......... .......... .........
AB481071.1|_JPOC06-007            .......... .......... ........-. .......... .......... .........
JF699044.1|IR-RIGLD-PA5176        .......... .......... ........-. .......... .......... .........
MF977917|Taif-4                   .......... .......... ........-. .......... .......... .........
MF977918|Taif-5                   .......... .......... ........-. .......... .......... .........
MF977919|Taif-6                   .......... .......... ........-. .......... .......... .........
MF977920|Taif-7                   .......... .......... ........-. .......... .......... .........
MF977921|Taif-8                   .......... .......... ........-. .......... .......... .........
MF977922|Taif-9                   .......... .......... ........-. .......... .......... .........
MF977923|Taif-10                  .......... .......... ........-. .......... .......... .........
MF977924|Taif-11                  .......... .......... ........-. .......... .......... .........
MF977925|Taif-12                  .......... .......... ........-. .......... .......... .........
MF977926|Taif-13                  .......... .......... ........-. .......... .......... .........
MF977927|Taif-14                  .......... .......... ........-. .......... .......... .........
MF977928|Taif-15                  .......... .......... ........-. .......... .......... .........
MF977929|Taif_16                  .......... .......... ........-. .......... .......... .........
MF977930|Taif-17                  .......... .......... ........-. .......... .......... .........
MF977931|Taif_18                  .......... .......... ........-. .......... .......... .........
MF977932|Taif-19                  .......... .......... ........-. .......... .......... .........
MF977933|Taif-20                  .......... .......... ........-. .......... .......... .........
MF977934|Taif_21                  .......... .......... ........-. .......... .......... .........
MF977935|Taif-22                  .......... .......... ........-. .......... .......... .........
MF977936|Taif-23                  .......... .......... ........-. .......... .......... .........
MF977937|Taif-24                  .......... .......... ........-. .......... .......... .........
GU048664.1|HBoV-2|CU1557UK        ......G... .....----- ---------- ---------- ---------- ---------
GQ200737.1|HBoV-2|KU1             ......G... .......... .....TT.CA .......G.. .......A.. .........
KM624025.1|HBoV-2|LZFB080         ......G... .......... .....TT.CT .GAGA----- ---------- ---------
FJ973558.1|HBoV2A-TU-A-114-06     ......G... .......... .....TT.CA .......G.. ......---- ---------
GQ867666.1|HBoV-3|MC8             ......G... .......... .....----- ---------- ---------- ---------
gFJ948861.1|HBoV-3|W855           ......G..- ---------- ---------- ---------- ---------- ---------
FJ973562.1|HBoV3B-TU-A-210-07     ......G... .......... .....TT.CA .....----- ---------- ---------
NC_012564.1|HBoV-3                ..C...G... .......... .....TT.CA .......G.. .......A.. .........
KJ649742.1|HBoV-4|RUS_NSC_11-N2657 ......G... .......... .....TT.CA .......G.. .......A.. .........
FJ973561.2|HBoV-4-NI-385          ......G... .......... .....TT.CA ..-------- ---------- ---------
KJ649741.1|HBoV-4|RUS_NSC_11-N2655 ..-------- ---------- ---------- ---------- ---------- ---------
```

Strains related to the HBoV-1 genotype are boxed.
Representative strains from HBoV-2, HBoV-3 and HBoV-4 are included.
